# Supplementary material for: UV-B irradiation promotes anthocyanin biosynthesis in the leaves of Lycium ruthenicum Murray
Source: PeerJ. 2024 Oct 14;12:e18199. doi: 10.7717/peerj.18199 (PMC11485054; doi:10.7717/peerj.18199)
Supplement: Supplemental Information 3 [file peerj-12-18199-s003.docx]

Supplementary information


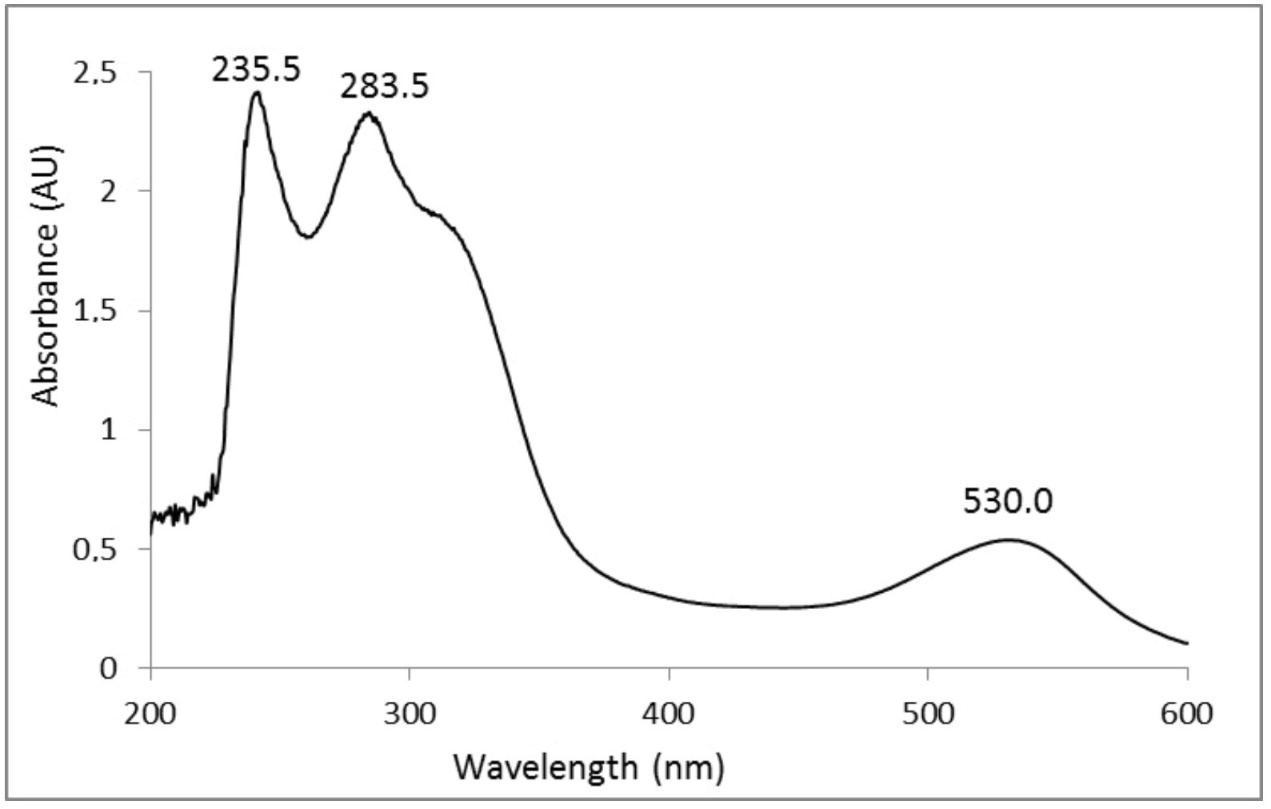


**UV-Vis spectra of the total anthocyanins ^[1]^**

[1]Demir, Taki & Gunesli, Zuhal & Sonmez, Fatih & Bilen, Cigdem & Yavuz, Emre & Gençer, Nahit. (2015). Inhibition of carbonic anhydrase I and II with total anthocyanins extracted from sweet cherry cultivars. Environmental Engineering and Management Journal. 14. 935-941. 10.30638/eemj.2015.104.
