# Supplementary material for: UV-B irradiation promotes anthocyanin biosynthesis in the leaves of Lycium ruthenicum Murray
Source: PeerJ. 2024 Oct 14;12:e18199. doi: 10.7717/peerj.18199 (PMC11485054; doi:10.7717/peerj.18199)
Supplement: Supplemental Information 5 [file peerj-12-18199-s005.docx]

Table S1 The primer sequences of RT-qPCR analysis

| **Unigene** | **Genename** | **ForwardprimerSequence(5’-3’)** | | **ReverseprimerSequence(5’-3’)** |  |
| --- | --- | --- | --- | --- | --- |
| Unigene0067978 | UVR8 | CTTGGGTTTCACCTACAGAGAG | CATCACTTTCCGGCACATTTAC | | |
| Unigene0008780 | COP1 | TGTACGCAGATCATAAGGGATG | GAGTTGGCTGGTAGTGAGATAA | | |
| Unigene0072293 | HY5 | GTATGGAGAGTGATGATGAGA | TTGCTTGCTGTGCTGATA | | |
| Unigene0010556 | CHS | GTGACACTCACTTGGATAGTATGG | GGCCTTTCAACCTCTGGTAAT | | |
| Unigene0001601 | UFGT | TCGGACATGTAGGGAACTAGAA | GATGATGGTTCGGGCAGTATAG | | |
| Unigene0070868 | MYB | CCCTAAAGAACCCGTGAATGT | GAAAGCGGAGAAGGCTCAATA | | |
| Unigene0025729 | bHLH | CCGGTACTGAAGGATTTGGTTAT | GTGGGCAGCTTGTAGTTCTT | | |
| Unigene0066868 | WRKY1 | GCCATCTCATTCCCACTCTT | GGCCAATTGCCTTATCCTTTG | | |
| Unigene0013893 | WRKY2 | ACAGGAAACCAAAGAGGATGT | AGACGATATAACATCTGGCGATAA | | |
| Unigene0039337 | WRKY3 | GAAGAGGACGCTACAAGAGAAG | CCTTGGATATTTGGCATTGAGG | | |
| Unigene0078996 | WRKY4 | GCTGATGCAGCTGTTACAAAG | CAGGATGAGGAGAAGCAATAGG | | |
| Reference gene | Actin | GAAGGGTGTCCCTCAGATCA | CCGTCCATGTCGTCTCTTTT | | |

Table S2 Base the information of reads statistics

| **Sample** | **RawData(bp)** | **BF_Q20**  **(%)** | **BF_Q30**  **(%)** | **BF_N**  **(%)** | **BF_GC(%)** | **CleanData(bp)** | **AF_Q20(%)** | **AF_Q30(%)** | **AF_N(%)** | **AF_GC(%)** |
| --- | --- | --- | --- | --- | --- | --- | --- | --- | --- | --- |
| Lr-CK-1 | 6154833600 | 5983252251 (97.21%) | 5689760370 (92.44%) | 131116  (0.00%) | 2654573084 (43.13%) | 6116808965 | 5953641816 (97.33%) | 5663623693 (92.59%) | 88467 (0.00%) | 2634988348 (43.08%) |
| Lr-CK-2 | 6178418700 | 6037724517 (97.72%) | 5777484075 (93.51%) | 135670 (0.00%) | 2670759405 (43.23%) | 6142829987 | 6008884554 (97.82%) | 5751662462 (93.63%) | 89433 (0.00%) | 2652189260 (43.18%) |
| Lr-CK-3 | 7319283000 | 7152996480 (97.73%) | 6852730314 (93.63%) | 88838 (0.00%) | 3138946413 (42.89%) | 7276032818 | 7118142712 (97.83%) | 6821456156 (93.75%) | 71633 (0.00%) | 3116495502 (42.83%) |
| Lr-UV-B-1 | 6146019600 | 5994444963 (97.53%) | 5725615797 (93.16%) | 134502 (0.00%) | 2682022117 (43.64%) | 6104716514 | 5961003628 (97.65%) | 5695798724 (93.30%) | 89795 (0.00%) | 2660429864 (43.58%) |
| Lr-UV-B-2 | 6168144600 | 6033167382 (97.81%) | 5784332704 (93.78%) | 123875 (0.00%) | 2740832097 (44.44%) | 6129184924 | 6001842882 (97.92%) | 5756379477 (93.92%) | 79325 (0.00%) | 2720389728 (44.38%) |
| Lr-UV-B-3 | 6233383500 | 6076149664 (97.48%) | 5791587475 (92.91%) | 68578 (0.00%) | 2713536139 (43.53%) | 6207888604 | 6057419489 (97.58%) | 5775380276 (93.03%) | 62674 (0.00%) | 2700197754 (43.50%) |

Table S3 BUSCO statistical results

| **Complete BUSCOs (C)** | **Complete and single** | **Complete and duplic** | **Fragmented BUSCOs (F)** | **Missing BUSCOs (M)** | **Total BUSCO groups** |
| --- | --- | --- | --- | --- | --- |
| 1145 | 1117 | 28 | 158 | 137 | 1440 |

| Table S4 Statistics of assembly quality | | | | | | | |
| --- | --- | --- | --- | --- | --- | --- | --- |
| **Genes Num** | **GC percentage** | **N50 number** | **N50 length** | **Max length** | **Min length** | **Average length** | **Total assembled bases** |
| 63698 | 38.3667 | 11343 | 1945 | 16915 | 201 | 1125 | 71723202 |


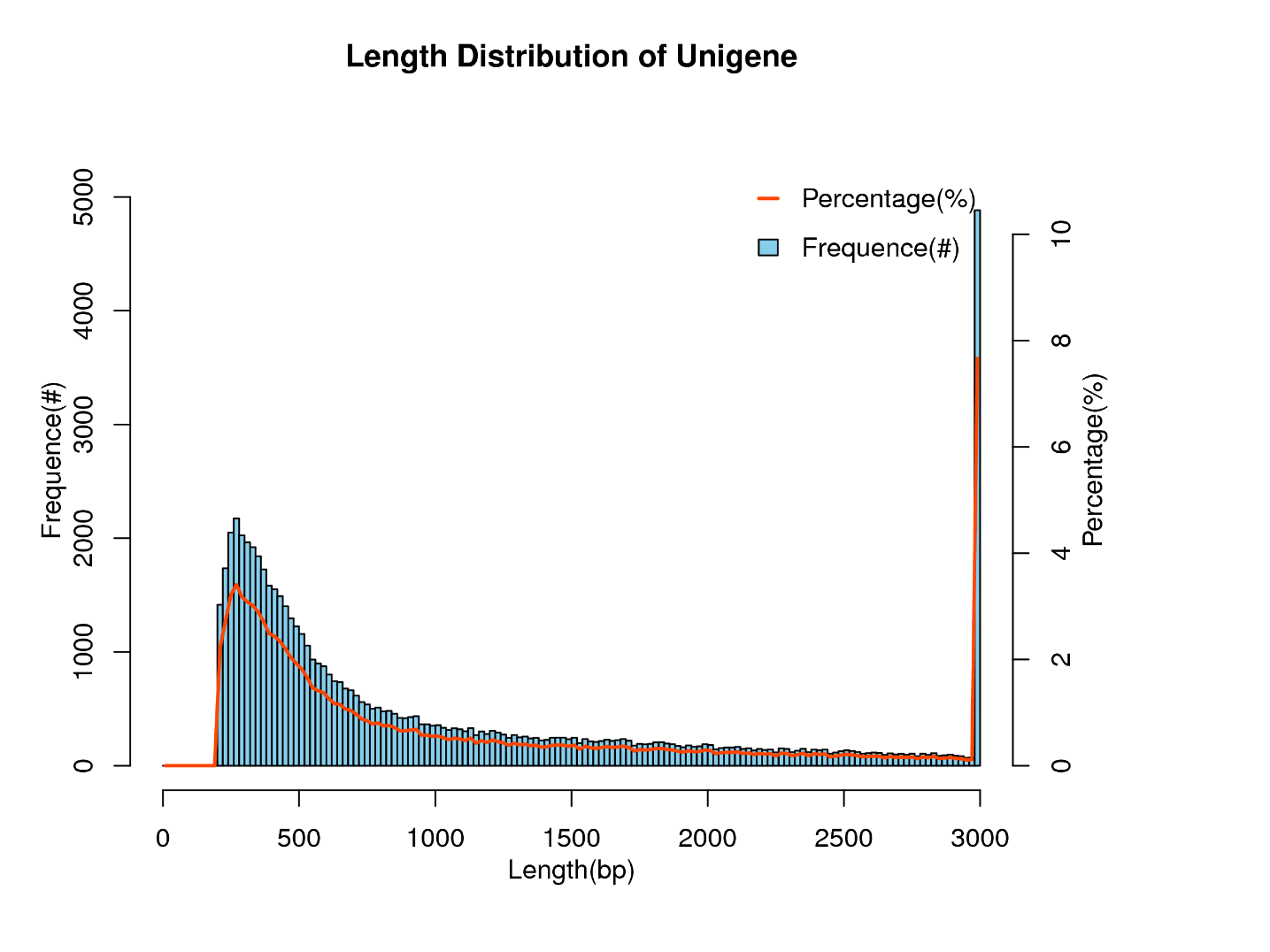


Fig S1 Length distribution of Unigene

Table S5 All Unigene annotation statistics

| Total Unigenes | Nr | KEGG_  with_KO | KEGG_with  Pathway | KOG | SwissProt | Annotation genes | Without  annotation gene |
| --- | --- | --- | --- | --- | --- | --- | --- |
| 63698 | 32130 | 11450 | 6064 | 16060 | 19711 | 32535 | 31163 |
