# Supplementary material for: UV-B irradiation promotes anthocyanin biosynthesis in the leaves of Lycium ruthenicum Murray
Source: PeerJ. 2024 Oct 14;12:e18199. doi: 10.7717/peerj.18199 (PMC11485054; doi:10.7717/peerj.18199)

Supplementary information for the MIQE checklist

1、Nucleic acid quantification:

Table1 Purity (A260/A280)

| Sample name | OD_260/280_ |
| --- | --- |
| LR-0-2-1 | 1.85 |
| LR-0-1-3 | 1.87 |
| LR-0-3-1 | 1.87 |
| LR-1-1-3 | 1.85 |
| LR-1-2-1 | 1.89 |
| LR-1-3-1 | 1.87 |
| LR-2-3-2 | 1.90 |
| LR-2-3-1 | 1.89 |
| LR-2-2-1 | 1.89 |
| LR-4-2-2 | 1.89 |
| LR-4-3-3 | 1.89 |
| LR-4-1-2 | 1.90 |

2、Electrophoresis traces：


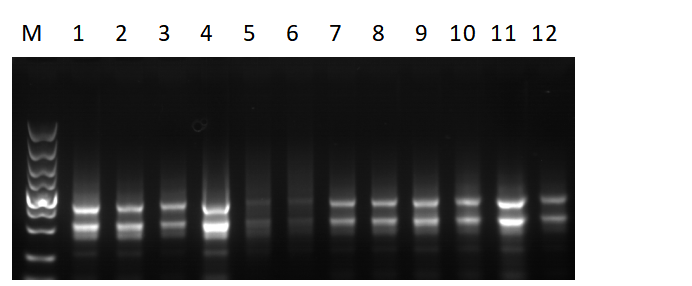


Figure 1 Agarose gel electrophoresis pattern (1.5% agarose, 180V, 15 min)

3、qPCR TARGET INFORMATION

（1）Sequence accession number

Table 2 Sequence accession number

| **Unigene** | **Gene name** | **Sequence accession number** |
| --- | --- | --- |
| Unigene0067978 | UVR8 | XP_019239275.1 |
| Unigene0008780 | COP1 | XP_004246103.1 |
| Unigene0072293 | HY5 | XP_016537585.1 |
| Unigene0010556 | CHS | AHH55328.1 |
| Unigene0001601 | UFGT | NP_001275017.1 |
| Unigene0070868 | MYB | KAH0708798.1 |
| Unigene0025729 | bHLH | KAF3657107.1 |
| Unigene0066868 | WRKY1 | KAG5622928.1 |
| Unigene0013893 | WRKY2 | XP_016436463.1 |
| Unigene0039337 | WRKY3 | XP_009586653.1 |
| Unigene0078996 | WRKY4 | PHT55134.1 |

（2）Location of amplicon (highlighted in green)

**UVR8：**

GCGGATGGGAATAGTGAAAGTATGTCCATTCGAAAAGTGCTTTTCATTTCCGCTGGTGCTAGTCACTCCGTTGCTCTTCTCTCTGGAAATGTAGTTTGCTCATGGGGAAGGGGAGAGGATGGACAACTCGGCCTTGGGGATGCTGAAGATCGATTTTCGCCGACTCAGGTGAGTGCATTGGATGGGCAGGAAATAGTATCTGTGACTTGTGGAGCTGATCATACAACTGCTTACTCGGAGGGCCTCAAGCAAGTCTATAGCTGGGGATGGGGTGATTTTGGGAGATTGGGTCATGGAAACTCCAGTGACTTGTTCACTCCTCAACCAATAAAAGCACTACACGGTATACGCATTAAGCAGATTGCATGTGGAGACAGCCATTGTCTTGCTGTGACCATGGAAGGTGAAGTGCAGAGTTGGGGACGGAATCAAAATGGCCAACTAGGCCTTGGCACCACTGAGGACTCTCTTGTACCTCGAAGGATTGAAGCTTTCAAGGGAATACCTGTAAAAATGGTTGCTGCTGGTGCCGAACATACAGCTGCTGTTACAGAAGATGGTGAACTTTATGGTTGGGGTTGGGGTCGATATGGTAATCTGGGGTTAGGTGACCGAAATGATCGCTTGGTTCCAGAAAAAGTTGCAGCTGATGTGGGAGAAAAGATGTTCATGGTTGCATGCGGTTGGCGGCATACTATCTGTGTCTCCTCTTCTGGTGCTTTGTATACATATGGTTGGAGCAAATATGGTCAACTAGGACATGGTGATTTTGAGGATCACCTTTTTCCTCATAAGGTTCAAGCTTTGCATGAAAGTTTTACTTGTCAGATATCAGGTGGTTGGAGACATACCATGGCACGCACTTCTGATGGAAAACTTTATGGTTGGGGTTGGAACAAGTTCGGACAAGTTGGTGTTGGTGACAGCTGTGATCATTGTTCCCCAGTGCAAGTGAAATTTCCGCATGACCAGAAAGTAATTCTGATTTCATGCGGTTGGAGGCACACACTTGCTGTTACAGAAAGGCAGAATGTCTTTTCCTGGGGAAGAGGTACAAATGGTCAGCTAGGTCATGGGGAGTCTGTTGATAGGTATGTCCCAAGGATTATAGAAGCGTTAAGTGTTGATGGATCAAGTGGACAACAAATTGGATCTTCGACTTTTGACCCATCAGCAGCTGAAAAATCTTGGGTTTCACCTACAGAGAGATATGCTGTTGTTCCAGATGAAAATCTGCAGGTGCCAAGGCAAACAGTGATTTCAGTAGGAGGGAACGGAAACGATGTAAATGTGCCGGAAAGTGATGTGAAAAGGATACGATTA

**COP1：**

TGGCTATTAGACAGGGAATTATTGTGCCCTATATGTACGCAGATCATAAGGGATGCTTTTTTAACAGCTTGTGGACATAGTTTTTGCTATATGTGTATAGTTACTCATCTTCATATCAAGAGTGATTGTCCCTGTTGTTCTCATTATCTCACTACCAGCCAACTCTATCCGAATTTCCTCCTTGATAAGGTT

**HY5：**

ATGCAAGAGCAAGCGACAAGTTCCATTGCAGCTAGTTCTATACCTTCAAGTAGTGAGAGATCTTCTAGCTCAGCTCTCCATCTTGAAGTCAAAGAAGGTATGGAGAGTGATGATGAGATAAGAAGAGTGCCAGAGATGGGTGGAGAAGCCACGGGAACCACCTCTGCTTCTGGCAGAGATGGAACTTCTACTGCCGGTCAACCTCAACCATCATCAGCTGCTGGCACTCAAAGGAAGAGAGGAAGAAGTCCTGCTGACAAAGAAAACAAGAGGTTAAAAAGGTTGCTGAGAAATAGAGTATCAGCACAGCAAGCAAGGGAGAGGAAGAAAGCATACTTAATAGATCTGGAAGCAAGGGTGAAAGAATTGGAAACAAAGAATGCAGAACTTGAAGAGAGGTTGTCCACTTTGCAAAACGAGAACCAAATGCTTAGACATATACTGAAGAACACAACAGCAGGTGCTCAGGAAGGGAGGAAG

**WRKY-1：**

ATGGACAAAGGATGGGGTCTTACCCTTGATAGTTCTTCTGATAAAGTTGGTTTCTTTAAAAATAAACCTGTTTTTGGTTTTAATTTAAGTCCAAGATTGAATCATATCAACGCCAGTGAAATGTTTTCCGGCTCGGCTGATGAGAGACGTGGCATGGTTAATGAAGTTGACTTCTTCTCTGAGAAGAAATCTATTGGTCTTGTTGTAAAGAAGGAAAATTCTCAGGTTGACAATTCTATGAGGACTGATTATGTTGTAAATACTGGTTTGCAACTTGTGACTGCTAACGCTGGAAGTGATCAATCAACAGTAGATGACGGGATTTCATCGGAATTACTTGAAGATAAACGACCTAAAAATGAGCAGTTGGCACAATTGCAAGCTGAGCTTGAAAGGATGAATGCTGAAAATCAACGTTTAAAAGGGATGCTTACTCAAGTTGGCAACAGTTATTCTGCACTTCAGATGCATCTTGTTACACTCATGCAAGAGCAGCAGCAACAACAGCAGCAGAACAACAGCAGCAGCAACTGATTTCAAGAACTGAAAGTGCACATGACCGTGAGGTTGTTGAAGTGAAGCCTGAAGAAAAGAAGCAAGAGAATAATGAGACCATAGTTCCAAGACAATTCATGGAGCTTGGTCCAAGTGGTTCCAAAGCTAATCTAATGGAGGAGCCATCTCATTCCCACTCTTCATCAGAAGAAAGAACGCTTTCTGGATCACCTCGCAACAATACGGAATTAATGTCAAAGGATAAGGCAATTGGCCGGGAAGAAAGTCCAGAATCTGAGAGTTGGGCACCAAACAAGGTGCCTAAATTGATGAATTCACCAAAACCTGTTGAGCAATCAACAGAAGCAACTATGAGGAAAGCTCGTGTCTCCGTCCGTGCCCGATCAGAAGCTCCCATGATTAGTGATGGTTGCCAATGGAGAAAATATGGTCAAAAGATGGCAAAAGGCAATCCGTGTCCACGTGCTTATTATCGTTGCACCATGGCTGTTGGTTGTCCCGTGCGCAAACAGGTACAAAGGTGTGCAGAAGACAGGACAATCTTAATAACAACATATGAAGGTACACATAACCATCCCCTACCACCAGCAGCTATGGCTATGGCATCCACAACCTCAGCAGCAGCAAACATGTTACTCTCTGGTTCCATGCCAAGTGCTGATGGCATGATGAATGCAAATTTCCTAGCAAGGGCCATGTTACCATGTTCATCTAACATGGCAACTATTTCAGCATCAGCTCCATTTCCTACTGTCACATTGGACCTAACAGCCCAAAATCCAAATGCTGCATTGGCTAATTATCATCAAAGAATTAATCCAGCTAGTAATCAGTTCCAATTTCCTTTACCAGCTGGACTTAATCACCCGAATTTCGTTGCTTCAATGTCAAGTCCACAAATGCCACAGGTTTTAGGCCAAGCTTTGTATAACCAATCAAAGTTTTCAGGTTTACAAGTTTCTCAAGACAATATTCACCGCCCCTCTTTTTCTCATGACACACTGTCCGCTGCCACAGCCGCCATTACTGCTGACCCCAACTTCACTGCC

**WRKY-2：**

ATGGAATCTCCCTTGCCGGAAAACTCATCGACTGATCTGCAAAGGGCAATCGAAGGGTTAATCCGTGGTCGTGAATTTACGCGACGACTAAAAGAGATTATTAAAAAACCTCTTGGTGGTGGTGAAGTTGCAACCATTAATATGGCTGAGGATTTAGTTGGGAAAATTATGGAGTCATTTTCCGAAACTCTCTCCGTGATAAACTCCGATGATGTTAGAGTCGTCGATACGCCTTTTTCCGATGTTGAGGTGAAGTCACCGGAAGATTATTCTAGTGGAAGTTGCAAGAATACTTCAACATTGAAAGATCGAAGAGGATGCTACAAGAGAAGGTGATTTCTTACTCTTTTAATTCAATTACTAGTCTTAGCAATAGTTGAATATCAAACTTTTCCCTAAGGGTATTTTAGTCTTTTGACGTGGATGGTGCTAAGTTATTTCCTGTTCTTATTGGTTGATTGGTTCTCTTTTAGTTAATTATTGTGAAGTTTGGTTGGATTCTTGATGCTTATTGACTATGTTATCCATAACACCAAATGCTAGAAATACAAAAAGGGCTGGTCCCTATCAAGGCTTGGTGATAAGATTGATTAAAATTTTTCATATAGTTAATTGTATTAAACTTGCAATAATCATAGATCTCACTATAAGAGAATGCCTGTAAATTACCATTACAGAAGCTGTTTATTTATGAAAATGTGTTCAAACTTTACATCTCTAATAAATATACGTCTATCCTAGTATACGTCACTAATTAAGTTGTGTTATTTTAGTTACAATTTTCTCATAAATGATTTGGAAAATATCAAATGCAGGAAAACTTCAGAATCAAACATAAAAGAATCCTCAGATTTGGTGGATGATGGTCATGCTTGGAGAAAATATGGACAAAAACAGATTCTCCATGCCACTTATCCAAGGCACTACTTTAGGTGCACCCACAAATATGATCAAAAATGTCAAGCAAGCAAACAAGTGCAGAAAATCCAAGATAATCCACTACTATACCGAACAACATACTATGGACATCACACGTGCAAAGTTTTTCCTAGACATTCTCAAATAATTTTGGGTTCGCCAATGGATGGAAATTCCAATTCCATTATACTTAGTTTTGATCAAAACAATTGCCATCCTAATTGTTACAATAATAATAATAATGTCCCTACTACATTTCCCTCAATAAAACAGGAAACCAAAGAGGATGTTTTCAGATTCTATCCTAAAATTGAAGATCAAAATCAATCATCAACCTCTGATTATTTTCTGCCAGCAACATTTGAAGCCTCCGGTGGACACGTGGCGGCAGCGTTATCGCCAGATGTTATATCGTCTGGGGTATACTCTTCTTGTACTACTACAAGCAATGATAATCTTGAGATTGGTATTGATTTTGAGGAGTGTCTTTGGAATTTTGATGGGTATTCT

**WRKY-3：**

ACATCGTCTGATCTGGAAAAGATAATAGGAGAGTTGAATCGCGGCCGTGAATTGACGTGCCGGCTAAGAGAAATGATAAAGAAACCAATAGAGGATGAAGATGAGCATAATATGTTGGCTGAGGATTTGGTGGGGAAAATATTGAGTTCATTTTGTGTTACTCTATCGATGCTAAGTTCTAATGAATGCAATGAAGTTTCTCAAGTTATGAAGTCAACGGAAGAGTATTCAATTGGTAGTTGTAAGTCTTCTTCATTCAAAGATCGAAGAGGACGCTACAAGAGAAGGAGAACTTTAGAGACGAGCATAAAAGAAACCTCAACTTTGGTAGATGATGGCCATGCTTGGAGAAAATATGGTCAAAAACAGATCCTCAATGCCAAATATCCAAGG

**WRKY-4：**

ATGGCTATAGAGCTCGTGATGAATTACATAAACACAAATAGCAATAACAGCACTAGCTTCATGTCCAATTTGGAAGAAAAAACAGTGGTTCAAGAAGCTACCTCGGGGCTTCAAAGTGTCGAAAAATTCATCAAATTATTGTCACATAGTCAAAAACAAGGGAATTCAGCTTCTTTCATAAAGCAAGAAAAATCGACCCCAATGGAGATTGAAATAGTGGCTGATGCAGCTGTTACAAAGTTCAAGAACGTAATTTCTCTTCTTGGTCGAAAGAGAACTGGCCATGCTCGATTTAGAAGAGCACCTATTGCTTCTCCTCATCCTGATAATTTCAAGAAGGATTATTATGTTGAAACAAAAGTTTATTGTCCTACACCAATTCAACAACTCCCTCCAAAAGCCTATGATCGTTATAATCATCAATTATGTCAAAATCCAAATGATGGGGTTGTTAAAAAGCAGGAATTGGTTACAAAGACGATTAATTTTTCGGAAATTTGTCGTTCAAATTCGTTCAATATGTCAACTTTAACAGGGGAAACAGAGAGTAAAAGCTTCCAGATTTCAAATATTTCTCAGGTCTCTTCAGGTGTAAAGCCTCCTTTGTCTTCTTCTTCTTCTTCCTTCAAAAGTAGAAAGTGCACTTTGTCGGATAATGACCTTTCTGGCAAGTGCAGTGGTTCCTCTGGTCAATGCCATTGCTCAAAGAGAAGGAAATTAAGACTTAAAAGGGTAGTTAGAGTTCCAGCAATAAGCATGAAGCTGTCAGACATCCCGCCGGATGATTATTCATGGAGGAAATATGGACAAAAGCCAATTAAAGGATCTCCACATCCAAGGGCATACTACAAATGTAGTAGCGTGAGAGGTTGTCCAGCACGTAAACATGTAGAAAGAGCTCTGGATGAACCAACGATGCTCATTGTTACGTACGAAGGCGAGCATAACCATTCCGTTTCTGTTGCTGAAACAAGTAGTCTCATTTTAGAGTCTTCC

**CHS：**

ATGGTCACCGTCGAGGAGGTACGAAAGGCACAACGTGCACAAGGTCCGGCCACCATCATGGCCATTGGCACAGCCACTCCTTCGAATTGTGTTGATCAAAGCACCTATCCTGATTATTATTTTCGAGTCACTAATAGTGAGCATATGACTGAGCTTAAGGAGAAATTTAAGCGCATGTGTGACAAATCTATGATTAAGAAGAGGTACATGCACTTAACTGAAGAAATCCTAAAAGAAAACCCCAATATTTGTGAATACATGGCTCCTTCTATTGATGCTAGGCAAGACATAGTGGTGGTTGAAGTGCCAAAACTTGGCAAAGAAGCAGCCCAAAAGGCCATCAAAGAATGGGGCCAACCCAAGTCCAAGATTACCCATTTGGTATTTTGCACCACTAGTGGGGTGGACATGCCTGGGGCCGACTACCAGCTCACTAAGCTTCTTGGGCTTCGACCCTCCGTTAAGAGGTTCATGATGTACCAACAAGGTTGTTTTGCTGGTGGTACTGTTATCAGATTGGCCAAGGACTTAGCCGAAAACAACAAGGGTGCTCGAGTCCTTGTTGTTTGCTCAGAGATCACTGCAGTTACTTTTCGTGGCCCAAGTGACACTCACTTGGATAGTATGGTTGGACAAGCCTTATTTGGGGATGGGGCAGGTGCACTCATTGTAGGTTCTGATCCATTACCAGAGGTTGAAAGGCCTTTATTCGAGCTTGTCTCAGCAGCCCAAACTCTGCTCCCAGACAGTGAAGGTGCTATCGATGGGCACCTTCGTGAAGTTGGGCTAACATTTCACTTACTCAAAGATGTTCCTGGATTGATCTCAAAGAACATTGAGAAGAGCTTGATGGAAGCATTCCAACCATTGGGCATTTCTGATTGGAACTCTCTCTTTTGGATTGCTCATCCAGGTGGGCCGGCAATTCTGGACCAAGTTGAACTAAAGTTGGCCTTAAAGCCCGAAAAACTTCGAGCTACAAGGCAAGTCTTGAGTGACTATGGAAATATGTCTAGTGCTTGTGTTTTGTTTATTTTGGATGAAATGAGGAAGGCCTCAGCCAAAGAAGGGCTTGGTAGCACTGGTGAAGGCCTTGATTGGGGTGTACTCTTTGGATTTGGGCCTGGGCTAACAGTTGAGACTGTTGTGCTCCATAGTGTCTCTACT

**UFGT：**

AAGTTACACATAGCAATGTTCCCATGGTTTGCACTTGGTCATATAACCCCATTTCTCCAT

CTTTCAAATGAACTAGCTAAAAGAGGTCATCAAATCTCATTTTTATGTCCGAAAAAAGCTCAAATCCAAGTAGAGAACACCAATTTTCATCCAAATCTCATCACATTTCATTCTCTTAAAATTCCTCATGTTGATGGCCTTCCACTAGGCACAGAAACAGCCTCAGATGTGCCCATTTTCCTAACATGTCACCTTGCCACGGCCTTAGACCAAATGCGCGATCAAATCAAAATCTTACTCGCGAATTTAAAACCGGACATTGTGTTCTACGATTTCGCTTATTGGGTACCGGATATTGCCTCTGAAATCGGGTTCAAGACTGTATGTTACAACGTCGTATGTGCAGCATCCATCGCGATAGCGTTAGTCCCCGCGAGAAAATCATGTACTGTTGAAGAAATGATGAAGCCGCCAAAAGGGTACCCTCTGTCACAAGTAGTGCTCCGCGAACACGAAGCACGTGGACTTTTCTCTGTATTTCAAGAATACGGTAGTGGTGTAACGTTTTTTGACCGTGTCACAACAGCATTGGCGCGATGTGATGCTATATCAATTCGGACATGTAGGGAACTAGAAGGGCCTTTTTGTGACTATTTGTCTAGCCAATATAACAAGCCCGTGTTTCTACCCGGGCCTATACTGCCCGAACCATCATCACAAGATCGTCTAGATGAAAAATGGGCTAATTGGCTCCAACAGTTCAGGCCAGGGTCCGTCCTGTTCTGTGCTTTCGGGAGCCAATTAGTTCTTGAAAAGGGTCAGTTTCAAGAACTCGTTTTAGGAATTGAGTCAACAAATTTACCCTTTTTGATCGCGGTAAAACCTCCATTAGGTGTCAAGACAGTCGAGGAAGCCCTGCCGGAGGGATTTGAAGAGAGGGCTAAAGGAAAAGGAATAGTATATGGAGATTTTGTGCCACAAATTGGGATTTTGAATCACTCTTCAATTGGGTGTTTTGTGAGCCATTGTGGGTTTGGGTCAATGTGGGAGTCTTTAATGAGTGAGTGTCAAGTTGTGCTTGTACCGCATTTGGGTGACCAAATCTTGAACACTAGGCTTTTGGCCAATGAGTTAAAAGTTGCTGTTGAAGTAGAAAGAGATGAAAATGGATGGTTCACAAAAGAGAATTTGTGTAAAACTATAAGAAGTGTGATGGATGAAGGGAGTGAATTAGGTGAACTGATCAAGAAAAATCATGTCAAGTGGAAAGAGACACTAACAAGGCCTGGATTCATGAGTGGTTATATTGAAGAGTTCATGAATAATTTGCACAAGCTCTAGAAG

**MYB：**

GGTGTTCCCTGGACAGAGGAAGAGCACCGGCTTTTCTTGATTGGCCTACAGAAATTGGGCAAAGGAGATTGGCGGGGTATATCACGAAACTTTGTGACATCAAGGACTCCCACCCAAGTAGCTAGCCATGCCCAGAAGTATTTTATTCGGCAGACTAATGCTTCTCGGAGAAAGAGAAGATCCAGTCTCTTTGACATTGTTGCTGACACGGCTACCGATGGTTCTCATCCACTTCCTGAAGAACAATTTATGCTCCCACCTAGAGCAATGGAAAGTGATAAGGAACAGTTAGCACCTTCTGCAACAAAAGCAATAGAAACTGCTTTTGCAGATTCTCTCCCTTCCTTAGATCTTTCTCTCAAGCCAGATTTTGAATCCATGGAAACCGCTCCAAGTGAACCTGTTGAAGAAACGAAACCAAATACAACAACCAGCCAGATTCCTTCAGTATATCCAGCATTCTTCCCAGCTTACATTCCAGTTCCATATCCATTCTGGCCATCAAATGCAATTCCGGTTCCTGAAGATAGAGGAGCAGAACCATCCCATCATCAGGTCGTCAAGCCAATTCCAACCGTCCCTAAAGAACCCGTGAATGTAGATGAACTCGTGGGAATGTCCCAGCTCACTTTAGCAGACGCTGGTTCTGGCCATATTGAGCCTTCTCCGCTTTCCCTAAAATTAACAGCAGAACCATCAAGGCAATCAGCTTTTCATGCTAGCACACCAGTCAAAAGCTCCGAGATTACCAAGGGCGAAACTGCTCCAATTCAAGCACTT

**bHLH：**

ATGAATATTGCATTACCCGAAATGTTACACAGTATCACCGGCAATGGAAGCTCAGAAATGACCGTGCTAGAGCGACAACGAGCGAGAATGAAATGGCAACAGCAACAAGAGATGAGTTATTTTAATGAACAAAATGATCAACTGATGAATTCTTTTCATCACACGTCTGAAGCTCAACAATTTCATGGTCTGATCAATGTAAACGATCAGAGTCTTAGTGAGCTTGTGACTCGGGCAATTAAGTCAGACCCCAGTTTAGAGAACAATTGGGGTGATTTTGGGAACACCGGTACTGAAGGATTTGGTTATGTTTCAGTTGGGGTTGGACATGGAGGAATGTCACACCCAAGTGAAATGAATTATGCGATTTCAAGAACTACAAGCTGCCCACCTACCATGGCGGAAAATGCCGTTTCTGCTGTTAAAGCCAAAGAGACTATCCTGAGTTCTAATAGAGGCAGGGAGAGCTTCAAGAAGAGAAAAGCAGATAAGAATCAACATCTCAAGGAGGTTGCAGAAGAGGAAACCAAAGACAAGAAATTGAAAGAATACGTAGAGGAGGGAGATTCCAAGGTAACAACAGAGAAATACAGCAACAAAAGGAGCGCCAGTAACAGTAACAACAGTAAGGAAACCTCTGATACTTCAAAGGAGAAGTCCAAAATTACTGAAGATAAAAAGCCTGACTATATTCATGTCAGAGCACGTCGGGGTCAAGCCACTGATAGCCACAGTTTAGCTGAAAGAGTAAGAAGGGAAAAGATCAGTGAAAGAATGAGATTTCTACAAGATTTAGTACCAGGATGTAACAAGATCACAGGGAAAGCAGGAATGCTAGATGAAATAATTAATTACGTCCAGTCTCTCCAAAGACAAGTAGAGTTCCTATCAATGAAACTGGCTGCTGTTAATCCAAGGCTTGATATCGACGTAGACAATTTCTTCAACAAAGATATATTTGCAACTAGCACGTGTAGTTTTCCCACAGTGGGAGCTGGAACACCATCCGAAATGCTTAGTATGGCCCCACGCCAGTTCAATTCATTGCAGCAAATAGTGTCAAGTTCTGGATCGGAAATGGGTATTCTAAATCTTAGAGAAATGGCGCTACGTAGAACCACTAGCGCTCCCGTACCAAACCCTGAACTATTTCTTGATTCATCCGGTATCAATGTAATT

**4、REVERSE TRANSCRIPTION**

Table 3 Amplicon length

| **Unigene** | **Genename** | **ForwardprimerSequence(5’-3’)** | **ReverseprimerSequence(5’-3’)** | **Amplicon length** | |
| --- | --- | --- | --- | --- | --- |
| Unigene0067978 | UVR8 | CTTGGGTTTCACCTACAGAGAG | CATCACTTTCCGGCACATTTAC | | 120 |
| Unigene0008780 | COP1 | TGTACGCAGATCATAAGGGATG | GAGTTGGCTGGTAGTGAGATAA | | 132 |
| Unigene0072293 | HY5 | GTATGGAGAGTGATGATGAGA | TTGCTTGCTGTGCTGATA | | 219 |
| Unigene0010556 | CHS | GTGACACTCACTTGGATAGTATGG | GGCCTTTCAACCTCTGGTAAT | | 100 |
| Unigene0001601 | UFGT | TCGGACATGTAGGGAACTAGAA | GATGATGGTTCGGGCAGTATAG | | 102 |
| Unigene0070868 | MYB | CCCTAAAGAACCCGTGAATGT | GAAAGCGGAGAAGGCTCAATA | | 96 |
| Unigene0025729 | bHLH | CCGGTACTGAAGGATTTGGTTAT | GTGGGCAGCTTGTAGTTCTT | | 105 |
| Unigene0066868 | WRKY1 | GCCATCTCATTCCCACTCTT | GGCCAATTGCCTTATCCTTTG | | 95 |
| Unigene0013893 | WRKY2 | ACAGGAAACCAAAGAGGATGT | AGACGATATAACATCTGGCGATAA | | 148 |
| Unigene0039337 | WRKY3 | GAAGAGGACGCTACAAGAGAAG | CCTTGGATATTTGGCATTGAGG | | 128 |
| Unigene0078996 | WRKY4 | GCTGATGCAGCTGTTACAAAG | CAGGATGAGGAGAAGCAATAGG | | 106 |
| referencegenes | Actin | GAAGGGTGTCCCTCAGATCA | CCGTCCATGTCGTCTCTTTT |  | |

**5、qPCR PROTOCOL**

Complete thermocycling parameters:

Stage 1：pre-denaturation

Number of Cycle：1

Stage 2：PCR reaction

Number of Cycle：40

95℃ 3sec

60℃ 30sec

**6、qPCR VALIDATION**

Specificity (gel, sequence, melt, or digest)

**ACTIN：**
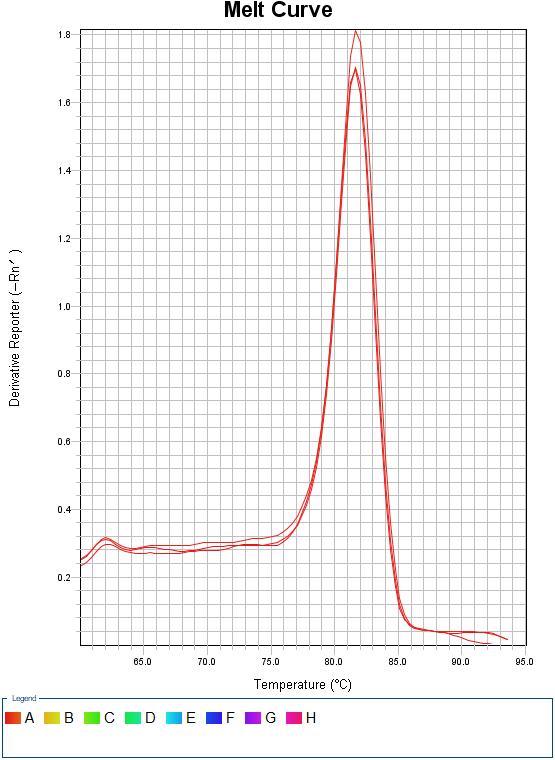


**HY5：**


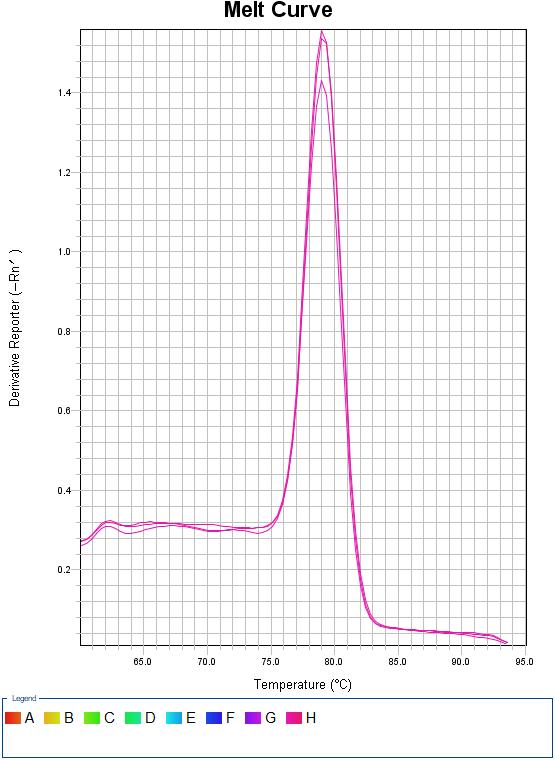


**bHLH：**


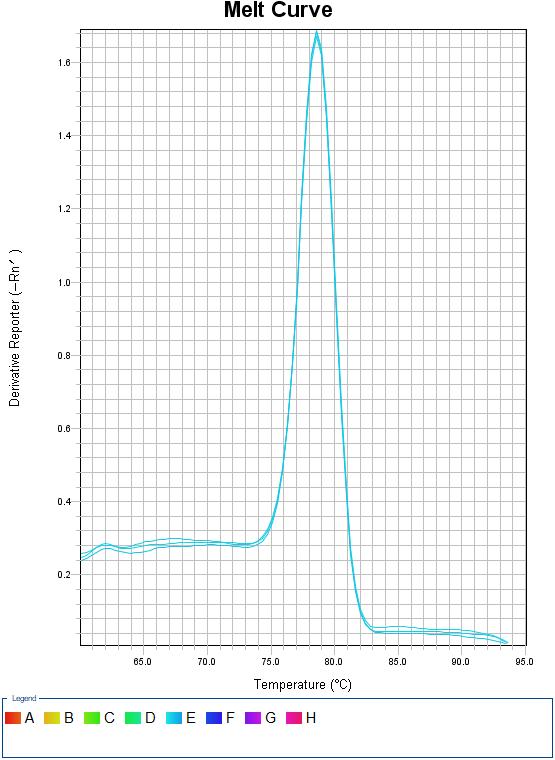


**CHS：**


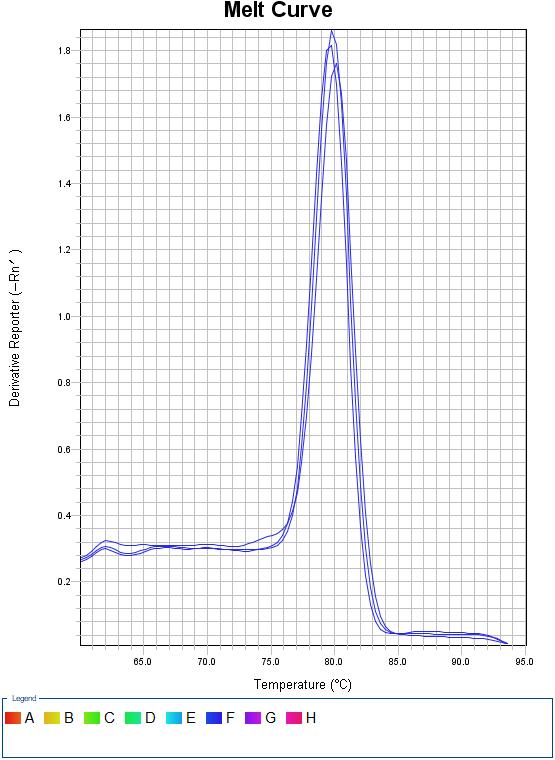


**COP1:**


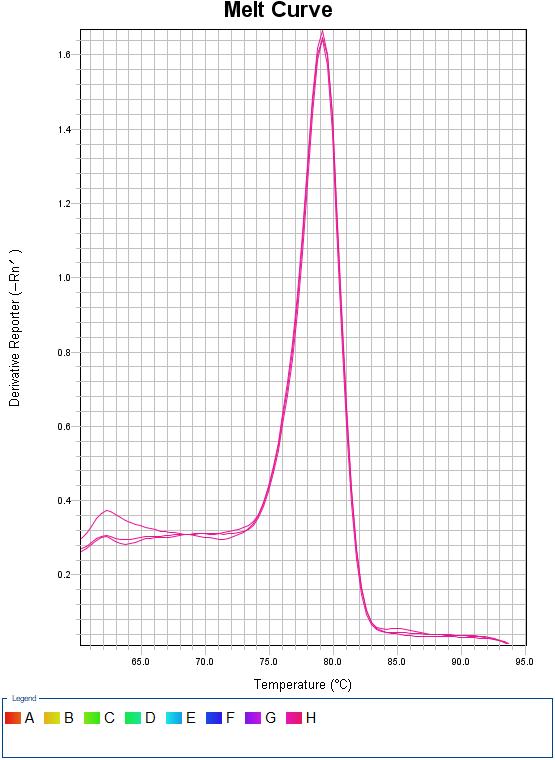


**UFGT:**


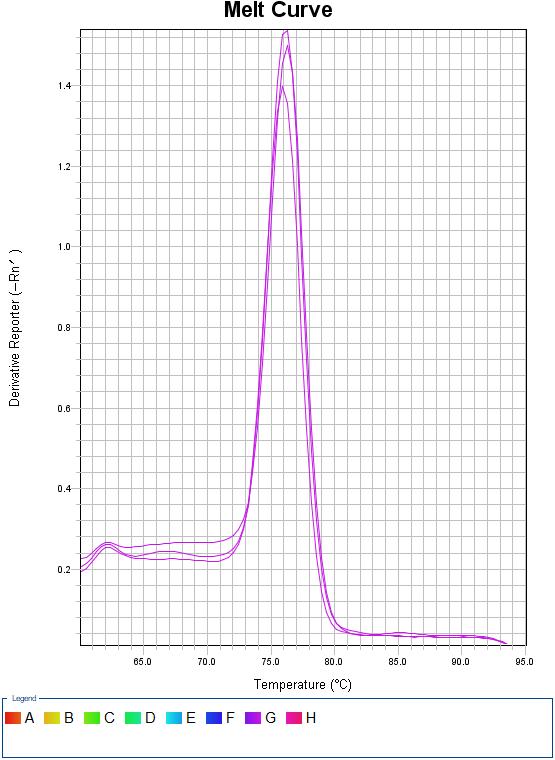


**MYB:**


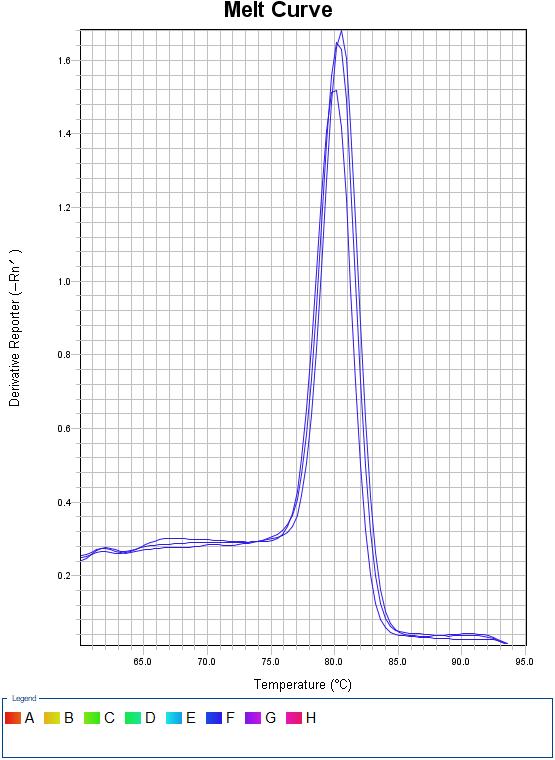


**UVR8:**


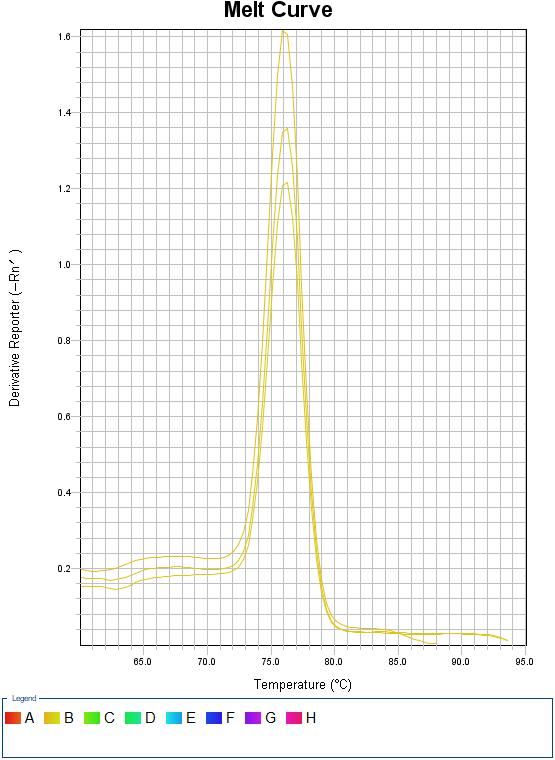


**WRKY1:**


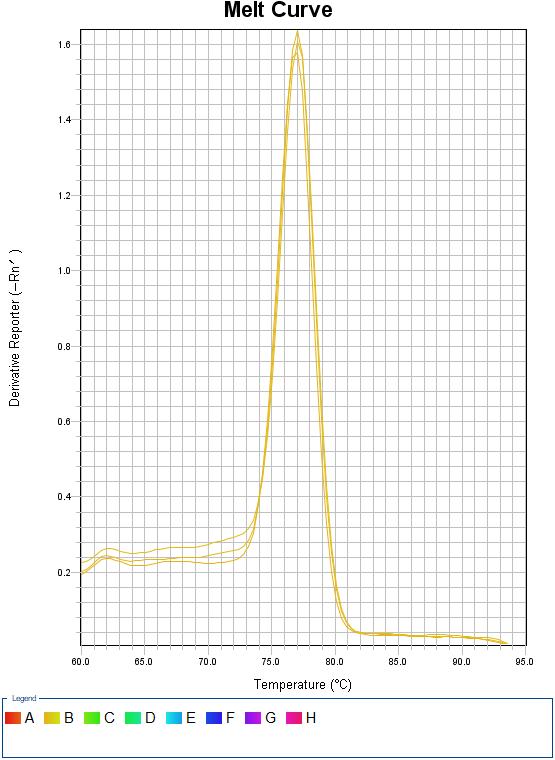


**WRKY2:**


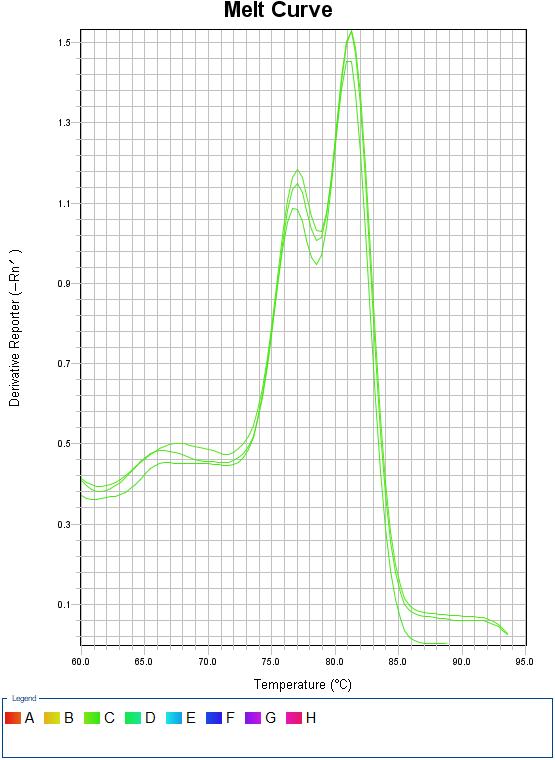


**WRKY3:**


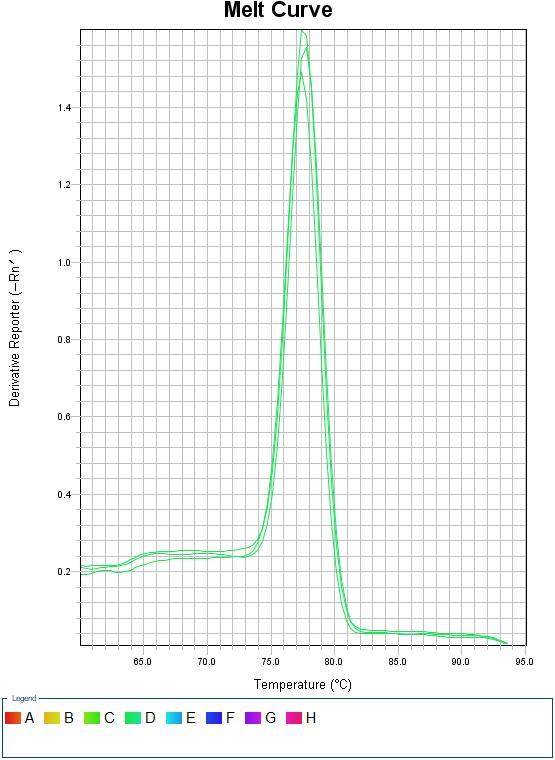


**WRKY4:**


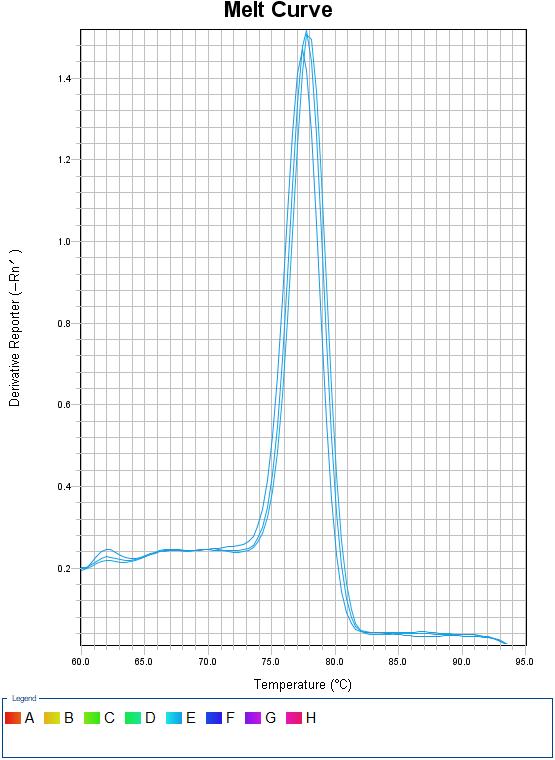

Supplement: Supplemental Information 9 [file peerj-12-18199-s009.docx]
